# Climate-driven variability of the Southern Ocean CO_2_ sink

**DOI:** 10.1098/rsta.2022.0055

**Published:** 2023-06-26

**Authors:** N. Mayot, C. Le Quéré, C. Rödenbeck, R. Bernardello, L. Bopp, L. M. Djeutchouang, M. Gehlen, L. Gregor, N. Gruber, J. Hauck, Y. Iida, T. Ilyina, R. F. Keeling, P. Landschützer, A. C. Manning, L. Patara, L. Resplandy, J. Schwinger, R. Séférian, A. J. Watson, R. M. Wright, J. Zeng

**Affiliations:** ^1^ Centre for Ocean and Atmospheric Sciences, School of Environmental Sciences, University of East Anglia, Norwich NR4 7TJ, UK; ^2^ Max Planck Institute for Biogeochemistry, PO Box 600164, Hans-Knöll-Str. 10, 07745 Jena, Germany; ^3^ Department of Earth Sciences, Barcelona Supercomputing Center, Barcelona, Catalonia, Spain; ^4^ Laboratoire de Météorologie Dynamique/Institut Pierre-Simon Laplace, CNRS, Ecole Normale Supérieure/Université PSL, Sorbonne Université, Ecole Polytechnique, Paris, France; ^5^ Department of Oceanography, University of Cape Town, Cape Town 7701, South Africa; ^6^ SOCCO, Council for Scientific and Industrial Research, Cape Town 7700, South Africa; ^7^ Laboratoire des Sciences du Climat et de l'Environnement, LSCE/IPSL, CEA-CNRS-UVSQ, Université Paris-Saclay, 91191 Gif-sur-Yvette, France; ^8^ Environmental Physics, ETH Zürich, Institute of Biogeochemistry and Pollutant Dynamics and Center for Climate Systems Modeling (C2SM), Zurich, Switzerland; ^9^ Alfred-Wegener-Institut Helmholtz-Zentum für Polar- und Meeresforschung, Postfach 120161, 27515 Bremerhaven, Germany; ^10^ Atmosphere and Ocean Department, Japan Meteorological Agency, 1-3-4 Otemachi, Chiyoda-Ku, Tokyo 100-8122, Japan; ^11^ Max Planck Institute for Meteorology, Hamburg, Germany; ^12^ Scripps Institution of Oceanography, University of California, San Diego, CA, USA; ^13^ Flanders Marine Institute (VLIZ), Jacobsenstraat 1, 8400 Ostend, Belgium; ^14^ GEOMAR Helmholtz Centre for Ocean Research Kiel, Kiel, Germany; ^15^ Department of Geosciences and High Meadows Environmental Institute, Princeton University, Princeton, NJ, USA; ^16^ Bjerknes Centre for Climate Research, Bergen, Norway; ^17^ NORCE Norwegian Research Centre, Jahnebakken 5, 5007 Bergen, Norway; ^18^ CNRM, Université de Toulouse, Météo-France, CNRS, Toulouse, France; ^19^ College of Life and Environmental Sciences, University of Exeter, Exeter EX4 4RJ, UK; ^20^ Earth System Division, National Institute for Environmental Studies, 16-2 Onogawa, Tsukuba, Ibaraki 305-8506, Japan

**Keywords:** Southern Ocean, carbon sink, climate, oxygen, interannual, decadal

## Abstract

The Southern Ocean is a major sink of atmospheric CO_2_, but the nature and magnitude of its variability remains uncertain and debated. Estimates based on observations suggest substantial variability that is not reproduced by process-based ocean models, with increasingly divergent estimates over the past decade. We examine potential constraints on the nature and magnitude of climate-driven variability of the Southern Ocean CO_2_ sink from observation-based air–sea O_2_ fluxes. On interannual time scales, the variability in the air–sea fluxes of CO_2_ and O_2_ estimated from observations is consistent across the two species and positively correlated with the variability simulated by ocean models. Our analysis suggests that variations in ocean ventilation related to the Southern Annular Mode are responsible for this interannual variability. On decadal time scales, the existence of significant variability in the air–sea CO_2_ flux estimated from observations also tends to be supported by observation-based estimates of O_2_ flux variability. However, the large decadal variability in air–sea CO_2_ flux is absent from ocean models. Our analysis suggests that issues in representing the balance between the thermal and non-thermal components of the CO_2_ sink and/or insufficient variability in mode water formation might contribute to the lack of decadal variability in the current generation of ocean models.

This article is part of a discussion meeting issue 'Heat and carbon uptake in the Southern Ocean: the state of the art and future priorities'.

## 1. Introduction

The Southern Ocean CO_2_ sink represents about 40% of the global oceanic CO_2_ sink. Large decadal variations have been evidenced from observations [1–6], which are not related directly to changes in emissions of CO_2_ from human activities but rather to variable climate conditions and/or variable external forcings and their influence on the growth rate of atmospheric CO_2_ [7–9]. It is essential to better characterize and simulate correctly the variability of the Southern Ocean CO_2_ sink in process-based ocean models to improve our understanding of the global carbon-cycle and its future evolution [9–13].

Two types of approaches widely used to estimate the oceanic CO_2_ sink and its spatio-temporal variability include ‘pCO_2_ products’, which are based on observations compiled in the Surface Ocean CO_2_ Atlas (SOCAT) [14], and Global Ocean Biogeochemistry Models (GOBMs), which are based on simulating the carbon cycle and its response to observed climate variability and changes in atmospheric CO_2_ [15]. Results from both methods suggest a relative stagnation of the Southern Ocean CO_2_ sink in the 1990s [2] and its reinvigoration in the 2000s [3]. However, they strongly disagree on the magnitude of these temporal variations, which are below 0.04 PgC yr^−1^ in GOBMs and around 0.08–0.18 PgC yr^−1^ in pCO_2_ products [11,15]. While pCO_2_ products might overestimate decadal variability due to sparse and unevenly distributed data [16,17], independent constraints from atmospheric CO_2_ inversions tend to support larger variability compared with GOBMs [15].

Here, we make use of a temporal decomposition methodology and of observation-based air–sea O_2_ fluxes to gain further insights on the variability of the Southern Ocean CO_2_ sink. First, we isolate the climate-driven variability of the Southern Ocean CO_2_ sink, i.e. the part of the CO_2_ sink caused only by fluctuations in climate, and decompose it into its short-term interannual component (i.e. year-to-year) and its longer-term decadal/sub-decadal component. This temporal decomposition aims to better identify the issues and underlying processes [16,18,19]. Second, we compare the climate-driven variability of air–sea fluxes of CO_2_ and O_2_, from both observational products and GOBMs, recognizing that CO_2_ and O_2_ are affected by the same processes, but in different proportions. Indeed, both O_2_ and CO_2_ in the ocean are influenced by thermal processes (e.g. warming of the surface ocean) in similar ways, while they are both also influenced by non-thermal processes (e.g. biological photosynthesis and respiration, and ocean circulation) largely in opposite ways [20,21].

The overall objective of this study is thus to provide potential constraints on the nature and magnitude of the climate-driven variability of the Southern Ocean CO_2_ sink by examining the coherence between CO_2_ and O_2_ flux variability estimated by data products and ocean models. For this, we evaluate the ability of 10 GOBMs to simulate interannual and decadal variability in air–sea fluxes of CO_2_ and O_2_ inferred from observations, and examine the overall coherence between CO_2_ and O_2_ variability. We hypothesize that if observation-based air–sea fluxes of CO_2_ and O_2_ from completely independent methods suggest similar interannual and/or decadal variabilities, then these are true signals of climate-driven variability of the Southern Ocean CO_2_ sink that GOBMs should simulate.

## 2. Data and methods

This study focuses on the period 1985 to 2018 and is based on monthly gridded data of air–sea fluxes of CO_2_ and O_2_ in the Southern Ocean from GOBMs and observation-based products. The Southern Ocean is defined here as the ocean area south of 30° S. The total oceanic CO_2_ sink (Total Flux) can be described as:

2.1 $$\text{Total }\text{Flux}={\text{Flux}}_{\text{ant}}^{\text{ss}}+{\text{Flux}}_{\text{ant}}^{\text{ns}}+{\text{Flux}}_{\text{nat}}^{\text{ss}}+{\text{Flux}}_{\text{nat}}^{\text{ns}},$$

where Flux_ant_ and Flux_nat_ are the air–sea fluxes of anthropogenic and natural CO_2_, respectively. The superscript *ss* (steady state) denotes fluxes under unchanging climate conditions (on time scales longer than a year), whereas *ns* (non-steady state) denotes fluxes that are solely affected by changing climate conditions. Therefore, ${\text{Flux}}_{\text{ant}}^{\text{ss}}$ captures the effect of rising atmospheric CO_2_ alone on the ocean CO_2_ sink, ${\text{Flux}}_{\text{nat}}^{\text{ss}}$ captures the flux of natural CO_2_ in a constant climate and ${\text{Flux}}_{\text{nat}}^{\text{ns}}+{\text{Flux}}_{\text{ant}}^{\text{ns}}$ captures the climate-driven variability of the ocean CO_2_ sink.

### (a) Air–sea CO_2_ fluxes

Each of the 10 GOBMs used here comprises an ocean physical model coupled with a marine biochemistry module (table 1). Models are forced with observed atmospheric CO_2_ mole fraction, and winds and other weather conditions from atmospheric reanalysis datasets (called ‘atmospheric forcing’). The 10 GOBMs differ through their use of different ocean physical models, representation of biogeochemistry, forcing products, spin-up strategies and spatial resolutions [15], all of which influence model representation of CO_2_ and O_2_ fluxes.

**Table 1.** List of the GOBMs and pCO_2_ products used in this study with some of their characteristics.

GOBMs			
name	physical ocean model	biogeochemistry model	atmospheric forcing
CESM-ETHZ	CESMv1.3	BEC	JRA55
CNRM	NEMOv3.6	PISCESv2	JRA55
EC-Earth3-CC	NEMOv3.6	PISCESv2	JRA55
FESOM-REcoM	FESOM-1.4	REcoM-2	JRA55
MPIOM-HAMOCC	MPIOM	HAMOCC6	NCEP
IPSL	NEMOv3.6	PISCESv2	JRA55
NorESM1-OCv1.2	MICOM	HAMOCC	NCEP
MOM6-Princeton	MOM6-SIS2	COBALTv2	JRA55
NEMO-PlankTOM12	NEMOv3.6	PlankTOM12	NCEP
ORCA025-GEOMAR	NEMO-ORCA025	MOPS	JRA55

pCO_2_ products			
name	gas exchange parameterization	wind product	atmospheric CO_2_ fields
CMEMS-LSCE-FFNN	quadratic exchange formulation [22]	ERA 5	[23]
CSIR-ML6	quadratic exchange formulation [22]	ERA 5	NOAA
Jena-MLS	quadratic exchange formulation [22]	JMA55-do reanalysis	Jena CarboScope
JMA-MLR	quadratic exchange formulation [22]	JRA55	JMA-GSAM
MPI-SOMFFN	quadratic exchange formulation [22]	ERA 5	NOAA
NIES-NN	quadratic exchange formulation [22]	ERA 5	NOAA
OS-ETHZ-GRaCER	quadratic exchange formulation [24]	JRA55, ERA5, NCEP1	NOAA
Watson2020	Nightingale formulation [25]	CCMP	NOAA

All 10 GOBMs performed standardized simulations as part of the RECCAP2 project (https://reccap2-ocean.github.io/), following a common protocol (electronic supplementary material). Models conducted a simulation ‘A’ designed to capture the Total Flux term (equation (2.1)). For this, models were all forced with similar observed atmospheric CO_2_ mole fraction, and with variable weather conditions (electronic supplementary material, table S1). Models performed another simulation ‘C’ to capture the ${\text{Flux}}_{\text{ant}}^{\text{ss}}+{\text{Flux}}_{\text{nat}}^{\text{ss}}$ terms, where models were again forced with observed atmospheric CO_2_ mole fraction, but this time with weather conditions reflecting a climatological year (e.g. looping over the same year). The climate-driven variability ${\text{Flux}}_{\text{nat}}^{\text{ns}}+{\text{Flux}}_{\text{ant}}^{\text{ns}}$ was obtained by subtracting simulation C from simulation A.

All eight pCO_2_ products used here were part of the RECCAP2 project. Each product estimates the oceanic CO_2_ sink and its variability based on the observations of sea surface fugacity of CO_2_ (fCO_2_) from the SOCAT database [14]. First, fCO_2_ observations are interpolated and extrapolated in time and space using statistical or machine learning methods. Second, the air–sea CO_2_ fluxes are calculated by subtracting the corresponding atmospheric CO_2_ mole fraction from the monthly (or daily) gridded ocean fCO_2_ estimates, and multiplying the difference by a gas-exchange coefficient, which is a function of wind speed. The eight pCO_2_ products differ through their use of different methods to produce the gridded maps of fCO_2_, and in the variety of gas-exchange parametrizations and ancillary datasets required in the calculation of air–sea CO_2_ fluxes (table 1). The pCO_2_ products estimate the ‘Total Flux’ component of equation (2.1). In order to isolate the climate-driven variability component ${\text{Flux}}_{\text{nat}}^{\text{ns}}+{\text{Flux}}_{\text{ant}}^{\text{ns}}$, the model estimate of the air–sea CO_2_ fluxes driven by atmopsheric CO_2_ alone as captured by the multi-model average of simulation C was subtracted from the pCO_2_ products estimates.

For the remaining of the manuscript, air–sea CO_2_ fluxes refer to the climate-driven variability of the CO_2_ fluxes for simplicity, unless specified otherwise.

### (b) Air–sea O_2_ fluxes

All GOBMs provided monthly gridded air–sea O_2_ fluxes from their simulation ‘A’ except ORCA025-GEOMAR. Observation-based estimates are taken from an atmospheric inversion method that optimized air–sea fluxes of Atmospheric Potential Oxygen (APO) to track the observed changes in APO concentration [26]. APO combines atmospheric observations of O_2_ and CO_2_ (APO = O_2_ + 1.1 CO_2_) in a manner that is conservative with regard to land biosphere exchanges (−O_2_ : CO_2_ = 1.1 mol mol^−1^) [27]. The combustion of fossil fuels also influences APO. This contribution has been removed as part of the inversion method using fuel-specific O_2_ : CO_2_ ratios (globally weighted average −O_2_ : CO_2_ ≈ 1.4 mol mol^−1^). Therefore, APO data adjusted for the relatively well-known fossil fuel combustion are records of air–sea fluxes of O_2_ and CO_2_. It has been demonstrated that variability in APO air–sea flux is approximately equal to the variability in air–sea O_2_ fluxes [26,28]. Here, we assume that the variability in APO air–sea flux estimated by an atmospheric inversion provides an observation-based estimate of the variability in O_2_ air–sea fluxes.

The O_2_ air–sea fluxes are not influenced by a strong anthropogenic signal (i.e. Flux_ant_) as is the case for CO_2_ [26]. Following the principles of equation (2.1), the climate-driven variability in O_2_ air–sea fluxes is captured by the term ${\text{Flux}}_{\text{nat}}^{\text{ns}}$. Therefore, this observation-based estimate can be used as an independent constraint to evaluate the ability of GOBMs to correctly simulate the oceanic processes that influence air–sea exchanges of O_2_, which impact the climate-driven variability of the CO_2_ sink as well.

Here, we use the atmospheric CarboScope APO inversion (https://www.bgc-jena.mpg.de/CarboScope), which is based on the TM3 atmospheric tracer transport model. The inversion optimizes APO observations at five stations (period 1994–2018) or nine stations (period 1999–2018). Observations are from the Scripps O_2_ program (https://scrippso2.ucsd.edu/). The temporal variations in air–sea O_2_ flux estimated with the APO inversion method depend mainly on the inversion configuration, the quality of the APO data and the number and location of stations [26]. As the inversion is based on atmospheric observations, it is totally independent of the pCO_2_ products, which are based on oceanic observations. Note that the spatial patterns of the O_2_ air–sea fluxes cannot be studied with the atmospheric inversion because of an insufficient number of sampling stations in the Southern Hemisphere to obtain robust estimates of finer-scale spatial features [26].

### (c) Time series decomposition

The interannual and decadal/sub-decadal (hereafter named decadal) components of the climate-driven CO_2_ and O_2_ air–sea fluxes time series were isolated using the following signal decomposition methodology: (i) the long-term mean was removed to focus on the temporal variability, (ii) the seasonal cycle was removed by applying a 12-month moving average, (iii) the decadal component of this de-seasonalized time series was obtained by filtering this time series with a 48-month Hanning window, (iv) the interannual component was extracted by removing the decadal component from the original de-seasonalized time series, and (v) the interannual component was smoothed with a 5-month Hanning window to eliminate the small month-to-month variability. The Hanning window is a filtering function with a ‘bell-shaped’ curve used to smooth the signal by emphasizing the feature near the centre of the window. A 48-month wide window eliminates most year-to-year variability, and therefore isolates the decadal component. The significance of the correlation coefficients between two filtered time series takes into account the corresponding degree of freedom by considering the e-folding decay time of autocorrelation [29]. The standard deviation of each time series is used as a measure of the magnitude of the variability.

We apply similarly steps (i–v) above to time series of the Southern Annular Mode (SAM) index [30] and to the atmospheric forcing time series of wind speed and Sea Surface Temperature (SST) from the National Centers for Environmental Prediction (NCEP), after removing their long-term trends using a linear fit. However, for the SAM index, step (ii) was not applied because there is no clear seasonal cycle and step (v) used a longer 18-month Hanning window because the SAM index signal is noisier than the climate-driven CO_2_ and O_2_ air–sea fluxes time series.

## 3. Results

### (a) Overview of the recent changes in the Southern Ocean CO_2_ sink

On average over the year, the Southern Ocean is a sink for CO_2_. The CO_2_ uptake mostly occurs in the Subtropical Zone (between 30° S and the Subtropical Front) and the Subantarctic Zone (between the Subtropical and the Subantarctic Front). This is mainly driven by natural CO_2_ uptake due to the cooling of subtropical waters, which are transported southwards, and the anthropogenic CO_2_ uptake by recently upwelled waters with low anthropogenic CO_2_ concentrations, which are transported northwards [1,31]. In the Subpolar Zone (north of the seasonally ice-covered zone), there is a net outgassing of CO_2_ due to the upwelling of deep waters with a high concentration of dissolved inorganic carbon [1,31].

**Figure 1.**
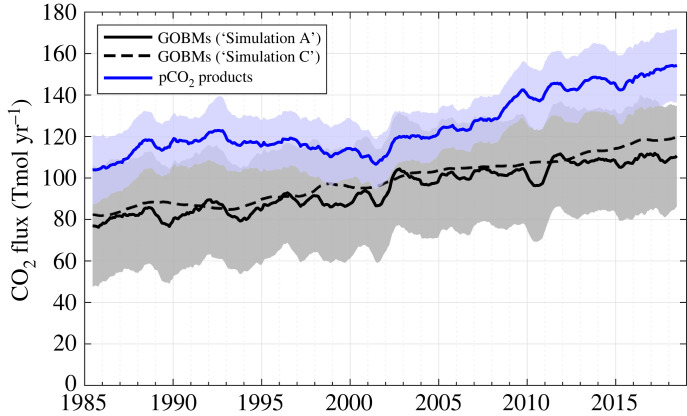
Southern Ocean CO_2_ sink from 1985 to 2019. Positive values denote a sink for CO_2_. The GOBM mean estimate (solid black line) is the average of 10 GOBMs with ±1 standard deviation of the model ensemble (grey shading). The dashed line represents the effect of increasing atmospheric CO_2_ only estimated from the GOBM mean. The pCO_2_ product mean estimate (blue line) is the average of eight pCO_2_ products with ±1 standard deviation of the product ensemble (blue shading). For the pCO_2_ product estimates, a river flux adjustment term of 31.6 Tmol yr^−1^ was added to be comparable with the GOBM estimates (see reference [15] for more details). The seasonal cycle was removed from all estimates with a 12-month moving average (see Methods). (Online version in colour.)

On average, the Southern Ocean CO_2_ sink increased by 30.6 Tmol yr^−1^ between the first and last decades (1985–1994 and 2009–2018, respectively) according to the pCO_2_ products (figure 1). GOBMs estimated a slightly lower increase of 24.5 Tmol yr^−1^, almost entirely caused by the response to the increasing atmospheric CO_2_ mole fraction (26.5 Tmol yr^−1^, dashed line in figure 1). Climate variability induced fluctuations in the Southern Ocean CO_2_ sink of approximately 6.9 Tmol yr^−1^ according to the pCO_2_ products and 3.3 Tmol yr^−1^ according to GOBMs, significantly larger than the fluctuations of about 1.9 Tmol yr^−1^ also estimated by GOBMs and caused by variability in atmospheric CO_2_ mole fraction only (estimated as the standard deviations of the detrended time series).

### (b) Simulated versus observed temporal variability of CO_2_ and O_2_ air–sea fluxes

**Figure 2.**
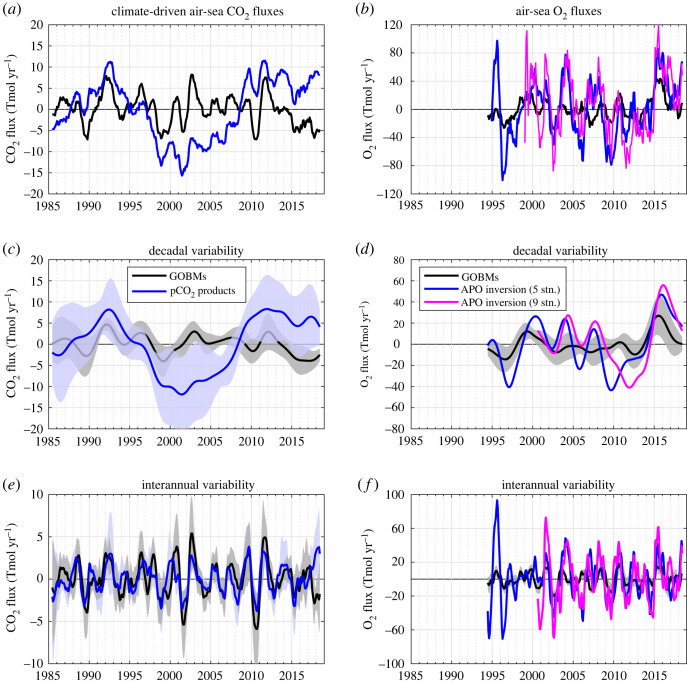
Comparison of the climate-driven air–sea CO_2_ (left) and O_2_ (right) fluxes estimated by GOBMs and observation-based products. (*a,b*) Climate-driven air–sea CO_2_ and O_2_ fluxes from GOBMs (black) and observation-based products (blue and magenta, see legend), decomposed into their (*c,d*) decadal and (*e,f*) interannual components, showing the mean across estimates and the ±1 standard deviation across the ensembles (shading). Fluxes are defined as positive from the atmosphere into the ocean. (Online version in colour.)

Temporal variations in the CO_2_ sink from the means of the GOBMs and pCO_2_ products are clearly different, both in phase and in magnitude (figure 2*a*). Most of the discrepancies are related to the amplitude of the decadal variability (figure 2*c*) which is uncorrelated and three times lower in GOBMs (2.0 Tmol yr^−1^) than in pCO_2_ products (6.3 Tmol yr^−1^). None of the 10 GOBMs simulates decadal variability that significantly correlates with that of the pCO_2_ product mean, and all GOBMs underestimate the magnitude of the pCO_2_ product mean by 24% to 77%. Moreover, all pCO_2_ products except one have a higher decadal amplitude than any other GOBM (table 2). However, only half of the individual pCO_2_ products are significantly and positively correlated with the mean pCO_2_ product signal, although the degree of freedom to test the significance of the correlation is small and some moderate or high positive correlation values (*r* ≥ 0.66) are considered non-significant. Longer time series are needed to increase the degree of freedom.

**Table 2.** Correlation (r) and amplitude of the decadal and short-term interannual variability in CO_2_ and O_2_ air–sea fluxes. Estimates inferred from the pCO_2_ product mean and the APO inversion with five stations (the first two lines), are compared with the fluxes inferred from each individual GOBM, the GOBM mean, each individual pCO_2_ product and the APO inversion with nine stations. The correlation coefficients are calculated with a Pearson correlation and take into account the degree of freedom for each time scale. Values in bold are statistically significant.

	CO_2_ (Tmol yr^−1^)				O_2_ (Tmol yr^−1^)			
	decadal		interannual		decadal		interannual	
	r (pCO^2^ product mean versus)	amplitude	r (pCO_2_ product mean versus)	amplitude	r (APO inversion (5 stn.) versus)	amplitude	r (APO inversion (5 stn.) versus)	amplitude
**pCO_2_ product mean**	—	6.31	—	1.68	—	—	—	—
**APO inversion (5 stn.)**	—	—	—	—	—	22.2	—	26.1
**GOBMs**								
CESM-ETHZ	0.08	2.8	**0**.**44**	2.4	**0**.**72**	8.5	0.28	9.9
CNRM-electronic supplementary material	−0.12	4.8	**0**.**5**	3.4	0.53	14.9	**0**.**36**	9.4
EC-Earth3	0.35	2.9	**0**.**62**	2.7	0.45	11.9	**0**.**41**	9.8
FESOM_REcoM	−0.02	1.9	**0**.**63**	3.0	0.55	5.8	**0**.**42**	9.5
MPIOM-HAMOCC	0.23	4.1	**0**.**58**	5.6	−0.07	15.4	**0**.**39**	16.0
NEMO-PISCES	0.08	3.1	**0**.**48**	2.3	0.55	10.6	**0**.**43**	9.6
NorESM	0.07	1.5	0.07	1.7	0.5	9.1	0.30	8.9
MOM6-Princeton	−0.05	3.6	**0**.**54**	2.7	**0**.**74**	8.2	**0**.**56**	10.0
NEMO-PlankTOM12	0.2	2.4	−0.32	2.1	**0**.**62**	16.6	0.20	11.3
GEOMAR	0.06	2.9	**0**.**57**	3.1	—	—	—	—
GOBM mean	0.12	2.1	**0**.**64**	2.1	**0**.**63**	8.5	**0**.**46**	8.3
**pCO_2_ products**								
CMEMS-LSCEFFNN	0.70	4.9	**0**.**72**	2.0	—	—	—	—
CSIR-ML6	**0**.**97**	6.7	**0**.**91**	2.1	—	—	—	—
Jena-MLS	0.46	8.0	**0**.**78**	4.9	—	—	—	—
JMA-MLR	0.66	5.8	**0**.**61**	1.6	—	—	—	—
MPI-SOMFFN	**0**.**95**	13.7	**0**.**55**	2.3	—	—	—	—
NIES-NN	0.63	2.7	**0**.**68**	1.2	—	—	—	—
OS-ETHZ-GRaCER	**0**.**94**	5.8	**0**.**79**	2.0	—	—	—	—
Watson2020	**0**.**96**	13.5	**0**.**66**	2.6	—	—	—	—
**APO inversion**								
APO inversion (9 stn.)	—	—	—	—	**0**.**79**	24.7	**0**.**68**	28.1

The decadal variability in O_2_ fluxes is also lower in the GOBM mean (8.5 Tmol yr^−1^) than in the atmospheric inversion (22.2 Tmol yr^−1^), but they are significantly correlated (*r* = 0.63, figure 2*d*). However, the correlation between the GOBM mean and the inversion is not improved when using the version of the atmospheric inversion with nine stations. Only three of the nine GOBMs have a significant correlation with the atmospheric inversion using five stations (0.62 ≥ *r* ≥ 0.74), and two with the inversion using nine stations (0.74 ≥ *r* ≥ 0.77). All GOBMs underestimate the magnitude compared with the inversion estimate by 25% to 74%.

The interannual variabilities in CO_2_ and O_2_ air–sea fluxes are more similar between the GOBM mean and the observation-based estimates (figure 2*e,f*). For CO_2_, the interannual variability is significantly correlated (*r* = 0.64) and similar in magnitude (2.1 Tmol yr^−1^ for GOBMs and 1.7 Tmol yr^−1^for pCO_2_ products). The median values of the interannual amplitude from all individual GOBMs and from all individual pCO_2_ products are similar (*p*-value = 0.0634; Wilcoxon Rank Sum Test). Eight GOBMs have a positive correlation with the pCO_2_ product mean (0.44 ≥ *r* ≥ 0.63). All individual pCO_2_ products are positively correlated with the pCO_2_ product mean (0.55 ≥ *r* ≥ 0.91; table 2). For O_2_, the interannual variability is also significant correlated (*r* = 0.46 and *r* = 0.55 for the inversions with the five and nine stations, respectively). Six of the nine GOBMs are significantly correlated with the atmospheric inversion (0.36 ≥ *r* ≥ 0.56). However, the magnitude of the mean GOBM interannual variability (8.3 Tmol yr^−1^) is three times lower than that estimated by the atmospheric inversion (26.1 Tmol yr^−1^). Within the observation-based estimates, the winter season is more correlated with the interannual variations (*r* = 0.97 for CO_2_, and *r* = 0.96 for O_2_) than the summer season (*r* = 0.87 for CO_2_, and *r* = 0.71 for O_2_).

### (c) Simulated versus observed spatial variability of CO_2_ and O_2_ air–sea fluxes

**Figure 3.**
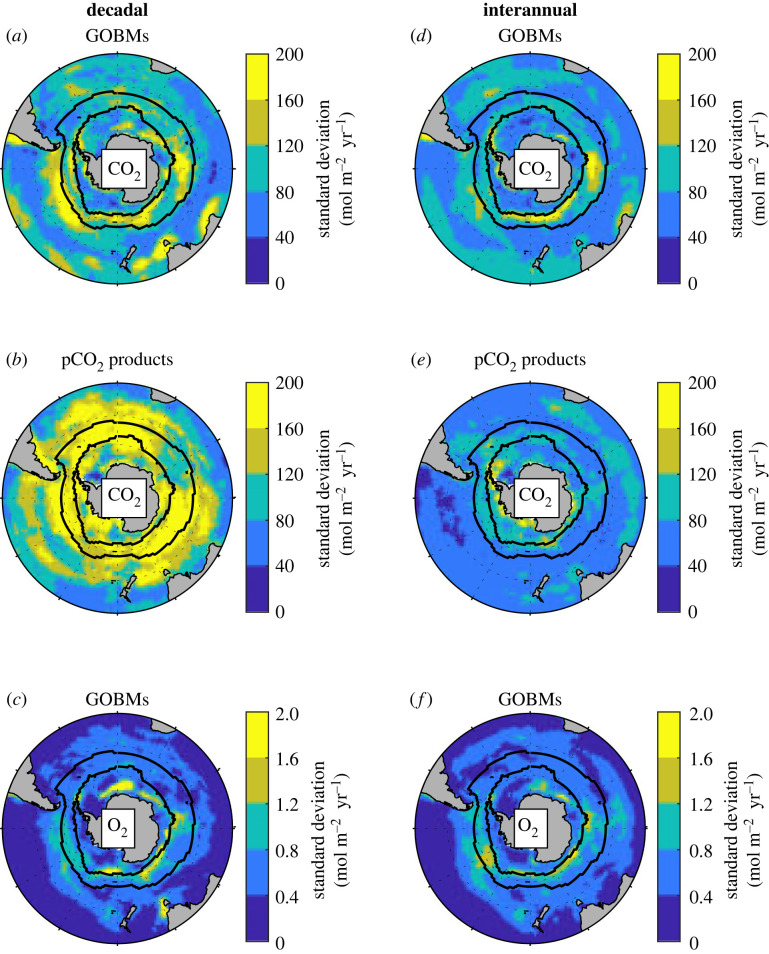
Standard deviation of the decadal (left) and interannual (right) variability of CO_2_ and O_2_ air–sea fluxes. For CO_2_ (*a,b* and *d,e*), the averaged maps from GOBMs and from pCO_2_ products are shown, while for O_2_ (*c* and *f*) only GOBMs could be used to study the spatial patterns. Note the differences in units and colour scales between O_2_ and CO_2_ maps. The black lines represent the average location of the Subantarctic Front (northern line) and of the September extent of sea ice (southern line). Maps from individual GOBM and pCO_2_ product are available in the electronic supplementary material (figures S1–S6). (Online version in colour.)

For the decadal component of the CO_2_ fluxes, there are clear differences between GOBMs and pCO_2_ products (figure 3*a,b*). First, the values are in general two times lower in GOBMs than in the pCO_2_ products. Second, in GOBMs, most of the highest magnitudes are south of or along the Subantarctic Front, apart from coastal regions south of Australia. In pCO_2_ products, areas south of the Subantarctic Front are also of importance, but they extend well to the north of this front and in all basins. For the decadal component of O_2_ air–sea fluxes in GOBMs, the areas south of the Subantarctic Front are of importance according to GOBMs (figure 3*c*).

For the interannual component of the CO_2_ fluxes, the areas of highest variability occur south of the Subantarctic Front in both GOBMs and pCO_2_ products (figure 3*d,e*). GOBMs highlight the importance of the Indian and Pacific sectors, a distinction less clear in the pCO_2_ products. For the interannual component of O_2_ fluxes (figure 3*f*), the area south of the Subantarctic Front is also of importance according to GOBMs, with also a larger influence of the Indian and Pacific Oceans, as for the decadal variability (figures 3*c,f*).

### (d) Relationship between observed variability of CO_2_ and O_2_ air–sea fluxes

**Figure 4.**
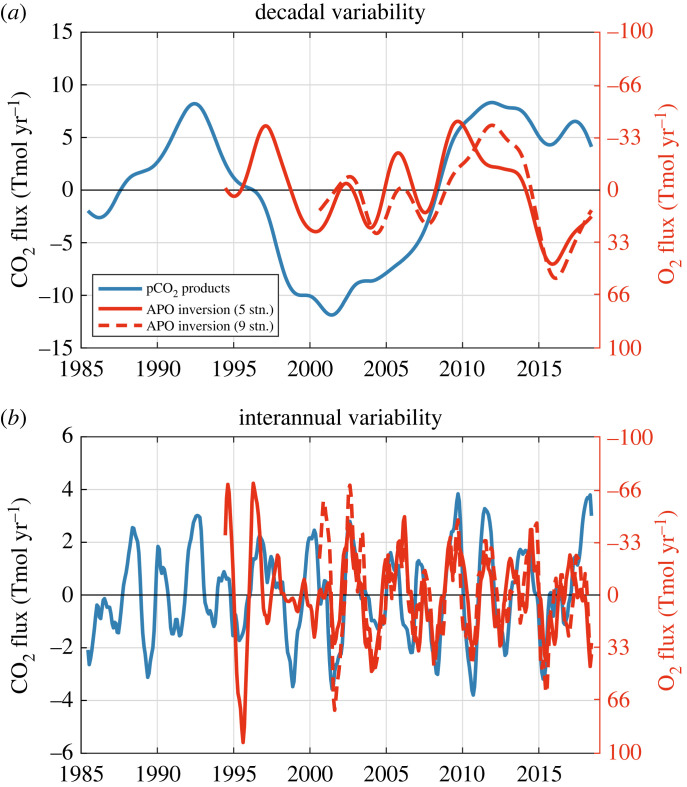
Comparison of the variability in CO_2_ and O_2_ air–sea fluxes. The (*a*) decadal and (*b*) interannual components of CO_2_ and O_2_ air–sea fluxes are from the observation-based products (i.e. pCO_2_ products and atmospheric inversion with five or nine stations). The y-axes on the right for O_2_ air–sea fluxes are inverted. Fluxes are defined as positive from the atmosphere into the ocean. (Online version in colour.)

The observed decadal variability in air–sea CO_2_ flux from the pCO_2_ product mean is significantly correlated with the decadal variability in air–sea O_2_ fluxes inferred with the APO atmospheric inversion based on nine stations only (*r* = –0.81, figure 4*a* and table 3), but not with the five-station inversion. This significant correlation is mostly induced by three individual pCO_2_ products that have correlation values *r* ≤ –0.72 (table 2).

**Table 3.** Correlation between observation-based variability of CO_2_ and O_2_ air–sea fluxes inferred from the pCO_2_ product mean and the APO inversion, respectively (with five or nine stations). The analysis was done with the decadal and interannual components (see also figure 4). The correlation coefficients are calculated with a Pearson correlation and take into account the degree of freedom for each time scale. Values in bold are statistically significant.

	decadal		interannual	
	APO inversion (5 stn.)	APO inversion (9 stn.)	APO inversion (5 stn.)	APO inversion (9 stn.)
**pCO_2_ products**				
CMEMS-LSCEFFNN	−0.03	−0.07	−0.1	**−0.37**
CSIR-ML6	−0.45	−0.69	**−0.41**	**−0.64**
Jena-MLS	−0.41	−0.62	**−0.38**	**−0.46**
JMA-MLR	−0.26	−0.08	−0.17	−0.34
MPI-SOMFFN	−0.58	**−0.72**	−0.25	**−0.55**
NIES-NN	−0.23	−0.37	−0.26	**−0.43**
OS-ETHZ-GRaCER	−0.31	**−0.76**	**−0.5**	**−0.62**
Watson2020	−0.41	**−0.75**	−0.25	**−0.43**
pCO_2_ product mean	−0.47	**−0.81**	**−0.4**	**−0.64**

The observed interannual variability in air–sea CO_2_ flux from the pCO_2_ product mean is significantly correlated with the interannual variability in air–sea O_2_ flux estimated with the APO atmospheric inversion with both five stations (*r* = –0.40) and nine stations (*r* = –0.64) (figure 4*b* and table 3). The significant correlation with the five-station inversion is supported by three out of the eight pCO_2_ products, while all pCO_2_ products except one support the significant correlation with the nine-station inversion (table 3).

Significant correlations between CO_2_ and O_2_ fluxes are all negative, suggesting that non-thermal processes, which influence CO_2_ and O_2_ in opposite direction, are the dominant source of variability on average over the Southern Ocean.

### (e) Relationships with the SAM index, wind speed and SST

**Figure 5.**
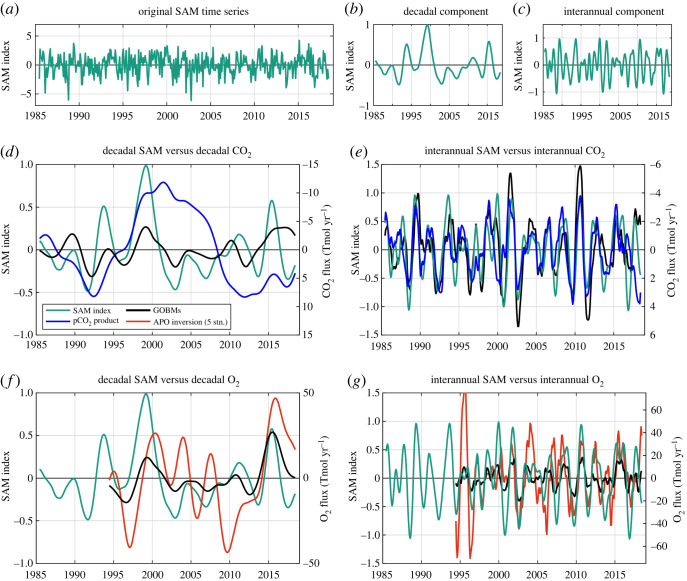
Comparison of the SAM index and the climate-driven variability of CO_2_ and O_2_ air–sea fluxes from the GOBM mean, pCO_2_ product mean and APO inversion. (*a*) The detrended time series of the SAM index, which has been decomposed into (*b*) a decadal component and (*c*) an interannual component, and compared with the decadal and interannual components from CO_2_ (*d* and *e*, the right-hand axes are inverted) and O_2_ (*f* and *g*) air–sea fluxes. Fluxes are defined as positive from the atmosphere into the ocean. (Online version in colour.)

On decadal time scale, significant correlations are found between the SAM and the CO_2_ and O_2_ air–sea fluxes from GOBMs (*r* = –0.52 for CO_2_ and *r* = 0.73 for O_2_), but not between SAM and the observation-based air–sea fluxes (i.e. pCO_2_ products and atmospheric inversion, figure 5*d,f*). Therefore, the simulated influence of the SAM on the decadal variability could be either spurious or due to missing or poorly represented processes in most GOBMs.

On interannual time scale, significant correlations are found between the SAM and the CO_2_ and O_2_ air–sea fluxes (figure 5*e,g*) in both models and observation-based fluxes (for GOBM fluxes, *r* = –0.71 for CO_2_ and *r* = 0.70 for O_2_; for observation-based fluxes, *r* = –0.56 for CO_2_ and *r* = 0.44 for O_2_). These results tend to support that the observed negative correlation between the interannual component of CO_2_ and O_2_ air–sea fluxes (see previous section) is also related to the SAM index.

**Figure 6.**
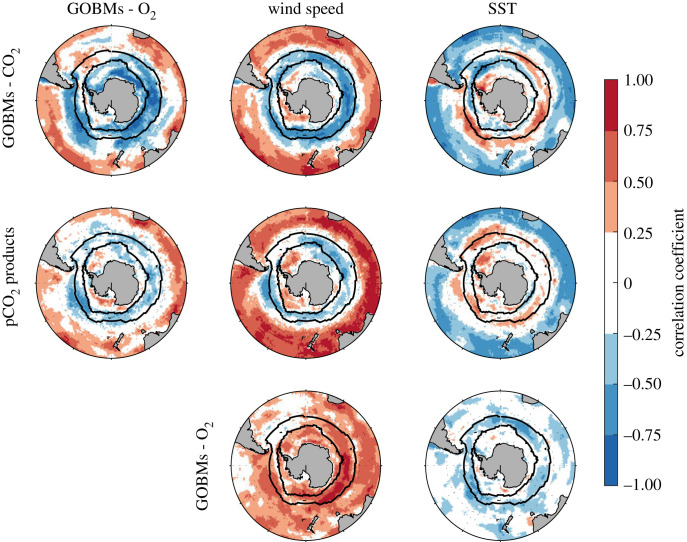
Correlation maps between the interannual components of the CO_2_ and O_2_ air–sea fluxes, and with the interannual components of wind speed and SST. The black lines represent the averaged location of the Subantarctic Front and of the September extent of sea ice. Fluxes are defined as positive from the atmosphere into the ocean. (Online version in colour.)

Negative correlations between CO_2_ and O_2_ fluxes on interannual time scale are associated with wind speed and occur in the Subpolar Zone (that extents from the Subantarctic Front to the September extent of sea ice; 15% of sea ice concentration, figure 6). In detail, in this region, correlations are mostly negative between CO_2_ fluxes and wind speed, and mostly positive between O_2_ fluxes and wind speed. Finally, and still in the Subpolar Zone, correlations are generally positive between CO_2_ fluxes and SST, and negative between O_2_ fluxes and SST. This suggests that interannual variations in the Southern Ocean CO_2_ sink are induced by processes occurring in the Subpolar Zone, with stronger CO_2_ outgassing events related to stronger winds, colder SST and stronger O_2_ ingassing events (and vice versa).

## 4. Discussion

The temporal variability of the Southern Ocean CO_2_ sink driven by climate variations can be decomposed into two components: a short-term interannual component and a decadal component (figure 2).

### (a) Interannual variability of the Southern Ocean CO_2_ sink

Our analysis suggests that pCO_2_ products accurately represent the climate-driven interannual variations of the Southern Ocean CO_2_ sink. This is supported by the strong similarities between the interannual variabily of CO_2_ and O_2_ fluxes provided by two completely independent observation-based products (figure 4*b* and table 3). Our analysis further suggests that this interannual variability is predominately regulated by wintertime deep-water ventilation south of the Subantarctic Front. This is supported (i) by the negative sign of the correlation between CO_2_ and O_2_ air–sea fluxes, which indicates the dominance of non-thermal processes [18], (ii) by the highest variability rates south of the Subantarctic Front, which corresponds to the location of ocean ventilation and outgassing of CO_2_ (ingassing of O_2_) [31,32], and (iii) by the higher correlation during the winter season pointing at a physical rather than a biological process. Therefore, the climatic processes regulating the wintertime ventilation of the Southern Ocean might also control the interannual variations of the Southern Ocean CO_2_ sink [33].

Our results indicate that the SAM could exert a strong control on the interannual variations of Southern Ocean CO_2_ sink (figure 5*e*,*g*). A positive (negative) SAM is related to an intensification (weakening) of the winds, which drive the upwelling intensity and carbon storage through the Ekman transport and other processes regulating the Mixed Layer Depth [2,34–36]. Years with stronger CO_2_ outgassing events in the Subpolar Zone are related to years experiencing stronger wind, colder SST (because of the upwelling of cold deep waters) and stronger O_2_ ingassing events (figure 6). Colder SST reinforces the ingassing of O_2_ in the Subpolar Zone (i.e. the thermal and non-thermal components reinforce each other), but dampens the outgassing of CO_2_.

Our results show that GOBMs simulate the interannual CO_2_ variability well, both in phase and in magnitude (figure 5*e*), including the SAM control of this variability. The correlation between the SAM index and the simulated and observed CO_2_ sink are similar for the interannual components (*r* = –0.64 and *r* = –0.51, respectively). This may be because the SAM is an atmospheric mode of variability and GOBMs are forced with atmospheric reanalysis dataset for wind forcing and surface heating whose variations are partly induced by the SAM.

The SAM is known to develop non-zonal atmospheric forcing in the Southern Ocean [37,38], inducing regional variations of the Mixed Layer Depth that influence the CO_2_ and O_2_ air–sea fluxes [18,19,32,39], but such regional features are currently missing in pCO_2_ products (figure 3*b*), possibly because of insufficient observational coverage (electronic supplementary material, figure S7). GOBMs simulated some regional variabilities, with a strong influence of the Indo-Pacific sectors on the interannual component of the CO_2_ sink (figure 3*a*). Recently, the Indo-Pacific sectors were pointed out as the main regions for CO_2_ outgassing when considering new indirect estimates of air–sea CO_2_ fluxes derived from Biogeochemical-Argo float observations [33,40]. New observations maintained over several years will be needed to confirm these regional influences [33].

Our findings are slightly conflicting with previous results that argued for the influence of the summer season on the short-term interannual variability of the CO_2_ sink [19]. However, this later study [19] only compares the year-to-year variability of monthly pCO_2_ during the months associated with the annual maximum and minimum of pCO_2_ and not the flux itself. In winter, small changes of pCO_2_ can be amplified by the strong wintertime winds. Our suggestion that wintertime deep water ventilation events have a key role also implies that variability in gas transfer velocity has a second-order effect on interannual variations of O_2_ air–sea fluxes, contrary to [41] who estimated that it was the most important process controlling the variability of O_2_ air–sea fluxes. However, their sensitivity analysis was done with model outputs instead of performing separate model simulations with non-varying piston velocity or deep-water ventilation. Nonetheless, they highlighted that changes in the ventilation of O_2_-depleted deep water strongly influenced the temporal variations of O_2_ air–sea fluxes south of the Subantarctic Front. Moreover, they also found a relationship between air–sea O_2_ fluxes and the SAM index and argued that it is driven by the influence of the SAM index on the upwelling rate of O_2_-depleted deep waters.

Although the processes controlling the interannual variability of CO_2_ and O_2_ air–sea fluxes are emerging from our analysis, uncertainties remain on their magnitude. Whereas the variability in the CO_2_ sink is comparable between GOBMs and pCO_2_ products, GOBMs underestimate the interannual variability in O_2_ air–sea fluxes by a factor of two to three, as pointed out elsewhere [26,41–43]. Further work is needed to examine the plausible causes of this discrepancy.

### (b) Decadal variability of the Southern Ocean CO_2_ sink

The decadal component of the air–sea O_2_ fluxes (figure 4*a*) tends to support the existence of a decadal climate-driven variability of the Southern Ocean CO_2_ sink. Longer time series of atmospheric APO will be needed before this can be firmly confirmed, but the existence of significant large decadal variability is consistent with several studies based on different methods (e.g. [5], see review in [1,9]). Decadal variations in the Southern Ocean CO_2_ sink are mostly marked by a saturation period in the 1990s [2] and a reinvigoration period in the 2000s [3]. According to the pCO_2_ products, the climate-driven decadal variations are three times larger than the interannual variations (figure 2).

Although our analysis does not fully resolve the magnitude of the decadal variability, it does suggest it is real and larger than GOBMs simulate, even though it may not be as large as estimated by pCO_2_ products due to uneven sampling [16,17]. The relative performance of the GOBMs in reproducing O_2_ decadal variability is better than that of CO_2_ decadal variability, suggesting that the representation of the balance between thermal and non-thermal processes might be partly at fault in models. The balance between thermal and non-thermal components of the CO_2_ sink has been shown to play an important role in driving seasonal [1,44] and interannual [6] variability. In contrast to CO_2_ fluxes, for which the thermal and non-thermal components oppose each other, the thermal and non-thermal components involved in the O_2_ air–sea fluxes reinforce each other [26,45], which might explain why some GOBMs were able to reproduce the observed decadal variability in O_2_ air–sea fluxes and less the decadal changes in the CO_2_ sink.

The spatial extent of the decadal variability in the air–sea CO_2_ flux north of the Subantarctic Front suggests that Subantarctic Mode Water formation might also influence the decadal variability in the Southern Ocean CO_2_ sink. Areas just north of the Subantarctic Front are where the upper cell of the Southern Ocean overturning circulation contributes to the subduction of mode waters, which could have substantial influence on the decadal variability of the CO_2_ sink. The subduction of surface water into intermediate layers was already mentioned to explain some of the regionally enhanced CO_2_ sink in the eastern Pacific between 2012 and 2016 [6]. Such subduction phenomena occur at specific locations in the Southern Ocean [46,47], which could explain the asymmetrical spatial pattern observed in pCO_2_ products. Subantarctic Mode Water is particularly important for the transport and recirculation of absorbed anthropogenic CO_2_ [48], but less so on its temporal variability, which is lower than the variability associated with natural CO_2_ [49]. However, variations in the uptake flux of anthropogenic CO_2_ could enhance the climate-driven variability of the natural CO_2_ flux [1]. For instance, an enhanced upwelling rate south of the Subantarctic Front could either slightly increase the CO_2_ sink north of this front by increasing the subsequent subduction of surface water that absorbed anthropogenic CO_2_, or slightly decrease this CO_2_ sink by shortening the residence time of the surface water [46,49,50].

The current generation of GOBMs have coarse resolutions and most likely do not correctly simulate the formation of Subantarctic Mode Water [51], which is sensitive to oceanic currents, wind speed, Mixed Layer Depth, sea ice and eddies [46,48,52]. Other studies highlight the importance of eddies after a positive SAM event that compensate for the intensified upwelling [53,54]. Therefore, improvements of the physical and ice ocean models might be needed to correctly simulate the decadal variability of the CO_2_ sink, as well as the future evolution in the Southern Ocean [55,56].

Other possible sources of variability missing in GOBMs include the coupling with atmospheric dynamics, internal tracer anomalies (memory) and ecosystem variability. Atmospheric coupling is unlikely to be driving large decadal variability of the Southern Ocean CO_2_ sink since Earth System Models also do not generate a ratio between the decadal and interannual variability similar to the one suggested by the pCO_2_ products ensemble mean [18]. Internal anomalies in dissolved inorganic carbon and/or O_2_ concentration could in theory trigger variability in air–sea fluxes when those anomalies become in contact with the atmosphere, but more work would be needed to verify if such anomalies in the ocean interior exist and how they relate to the patterns of variability identified here. Finally, changes in marine ecosystems in response to variability in ocean properties could act to enhance or dampen the thermal and/or non-thermal components and therefore amplify the total signals [5,34]. Current generation GOBMs represent ecosystems that are largely driven by upwelled nutrients and do not yet include the more complex ecosystem responses, such as vertical migrations and salps/krill dipole that characterize the Southern Ocean (e.g. [57]). The importance of these processes for the variability in CO_2_ and O_2_ fluxes has not yet been examined.

## 5. Conclusion

The degree of concordance between observed and simulated variability in air–sea fluxes of CO_2_ and O_2_, at different time scales, was used to gain insights on climate-driven changes in the Southern Ocean CO_2_ sink. The interannual variations of the Southern Ocean CO_2_ sink derived from pCO_2_ products and models are consistent with the interannual variations in air–sea O_2_ flux derived from observations. The current generation of GOBMs can simulate the influence of stronger (weaker) winds during years of positive (negative) SAM that induce, in the Subpolar Zone, stronger (weaker) upwelling of deep waters and drive the short-term interannual variations of the Southern Ocean CO_2_ sink. The decadal variations of the Southern Ocean CO_2_ sink, suggested by several pCO_2_ products, tends to be supported by the observed decadal variations of the air–sea O_2_ flux. However, GOBMs do not reproduce these decadal CO_2_ variations. Although the climate-driven processes associated with these decadal variations remain unclear, pCO_2_ products suggest an influence from regions associated with the formation of Subantarctic Mode Water, a physical process that might be poorly represented in GOBMs, while the relative performance of models in reproducing the decadal variability of O_2_ compared with CO_2_ suggests issues in representing the balance between thermal and non-thermal processes. More *in-situ* pCO_2_ data are required to confirm the influence of different Southern Ocean regions while more atmospheric APO data could help constrain the size of the decadal variability.

## Data Availability

All MATLAB scripts and data discussed in the results are publicly available. This GitHub repository contains instructions on how to access them: https://github.com/nmayot/PTA_SouthernOcean. The data are provided in the electronic supplementary material [58].
